# Supplementary material for: A Global Screen for Assembly State Changes of the Mitotic Proteome by SEC-SWATH-MS
Source: Cell Syst. 2020 Feb 26;10(2):133–155.e6. doi: 10.1016/j.cels.2020.01.001 (PMC7042714; doi:10.1016/j.cels.2020.01.001)

Q9Y2U5 | M3K2_HUMAN | MAP3K2 MAPKKK2 MEKK2
Monomer MW [kDa]: 69.741 Monomer expected elution fraction: 43

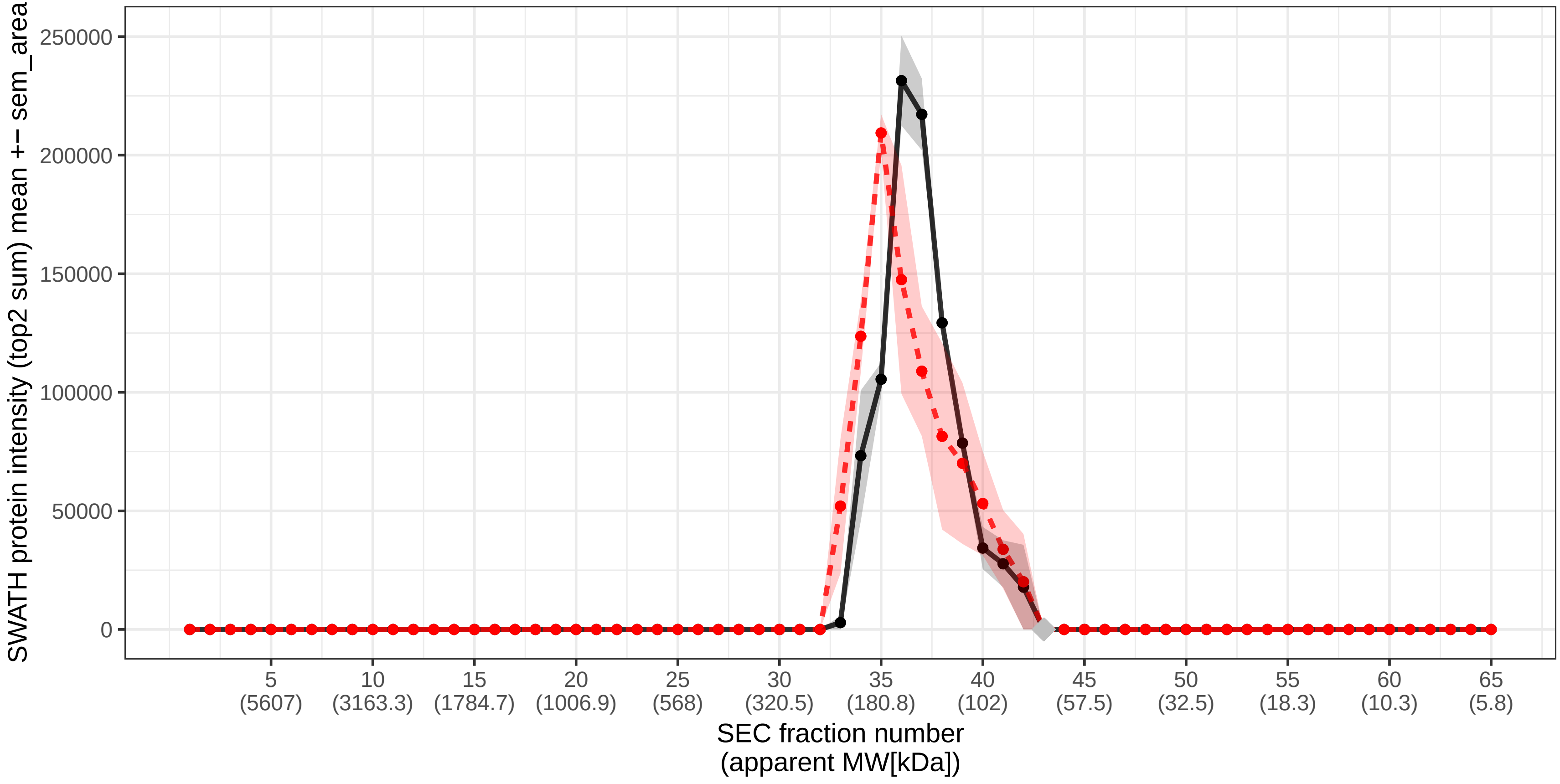

Supplement: Data S1. SEC-SWATH-MS Protein Chromatograms, Related to Figure 1 [file mmc6.zip › SECchrom_Q9Y2U5_M3K2_HUMAN_MAP3K2_MAPKKK2_MEKK2.pdf]
